# Design of a Solar Tracking System Using the Brightest Region in the Sky Image Sensor

**DOI:** 10.3390/s16121995

**Published:** 2016-11-25

**Authors:** Ching-Chuan Wei, Yu-Chang Song, Chia-Chi Chang, Chuan-Bi Lin

**Affiliations:** Department of Information and Communication Engineering, Chaoyang University of Technology, Taichung 41349, Taiwan; peter80810@gmail.com (Y.-C.S.); cblin@gm.cyut.edu.tw (C.-B.L.)

**Keywords:** solar tracking system, image sensor, brightest points, embedded processor, servo motor

## Abstract

Solar energy is certainly an energy source worth exploring and utilizing because of the environmental protection it offers. However, the conversion efficiency of solar energy is still low. If the photovoltaic panel perpendicularly tracks the sun, the solar energy conversion efficiency will be improved. In this article, we propose an innovative method to track the sun using an image sensor. In our method, it is logical to assume the points of the brightest region in the sky image representing the location of the sun. Then, the center of the brightest region is assumed to be the solar-center, and is mathematically calculated using an embedded processor (Raspberry Pi). Finally, the location information on the sun center is sent to the embedded processor to control two servo motors that are capable of moving both horizontally and vertically to track the sun. In comparison with the existing sun tracking methods using image sensors, such as the Hough transform method, our method based on the brightest region in the sky image remains accurate under conditions such as a sunny day and building shelter. The practical sun tracking system using our method was implemented and tested. The results reveal that the system successfully captured the real sun center in most weather conditions, and the servo motor system was able to direct the photovoltaic panel perpendicularly to the sun center. In addition, our system can be easily and practically integrated, and can operate in real-time.

## 1. Introduction

It is inevitable that human beings will face the exhaustion of fossil energy sources. Finding an alternative energy source that can satisfy global demand for energy is one of the most crucial and critical challenges that today’s society faces. The structures of the solar panels can be identified by the fixed structure and solar tracking structure. The solar tracking structure is not essential for the operation of a solar panel, but without it, the performance is reduced. Experiments show that photovoltaic (PV) panels with sun-tracking structures are able to generate 20%~50% more electricity than those with a fixed structure [1,2,3]. Solar tracking can be implemented by using one-axis, or for greater accuracy, two-axis sun-tracking systems. High-concentration photovoltaic (HCPV) systems can only accept direct solar light, and the acceptance angle deviation from the Sun will lead to a significant decrease in energy conversion efficiency. Therefore, a highly accurate and stable solar tracker is especially important to obtain maximum power for an HCPV system [4]. However, if the solar trackers can increase energy efficiency of PV arrays, some problems, such as cost, reliability, energy consumption, weather and maintenance may arise and counteract the benefits of the solar tracker.

Basically, solar-tracking systems are classified into two categories: passive (mechanical) and active (electrical) trackers [1]. Passive solar-tracking systems are based on the thermal expansion of chemicals (usually Freon) or on shape memory alloys. This kind of solar-tracking system is usually composed of a couple of actuators which work against each other. Under equal illumination, the solar-tracking system will be balanced. If the actuators are illuminated under different conditions, unbalanced forces will be generated to rotate the panel perpendicular to the point where the sun is located for realizing new equal illumination. 

Traditionally, active trackers can be classified as microprocessor and electro-optical sensor-based, PC-controlled date and time-based, auxiliary bifacial solar cell-based and a combination of these three systems. Nevertheless, from the viewpoint of control, the active solar-tracking systems can be realized as open loop or closed loop control systems [5,6]. Regarding an open loop control system, the trajectories of sun movement relative to the Earth can be precisely predetermined. Thus, there is no need for feedback to the tracking system. However, such systems need electronic and mechanical systems with high-precision, and lack the mechanism to automatically modify tracking errors [7,8,9]. Thus, if a solar power system is constructed on a mobile platform like vehicles and ships, the open loop control system will not be appropriate for satisfying the need for continuing modification of tracking parameters in such systems. With respect to the closed loop system, it usually uses photo sensors, such as light-dependent resistors or photodiodes, as feedback signals, and then sends these signals to control circuits to establish the approximate position of the sun [10,11,12]. The main disadvantage of using this type of sensor is the high sensitivity to temperature, humidity, and irradiation [13]. Additionally, cloudy days may severely affect the performance of the tracking system.

Recently, the use of low cost webcams as sensing elements for active solar tracking systems has been investigated [14,15,16]. Due to the mature technology of webcams and image processing, they can be easily adopted in practical solar tracking systems. Such tracking systems have also shown high efficiency in studies. Nevertheless, most of the image methods unavoidably used binary images to find the shape and position of the sun. The problem of finding the threshold value to generate the binary image arises. Different choices of the threshold value will lead to entirely different results. Many complex methods used to find the threshold value are presented, such as histogram shape, measurement space, clustering, entropy, object attributes, spatial correlation, and local gray-level surface [17]. Actually, to get the proper threshold value is complex and energy-consuming. Especially on cloudy days, the accuracy is low. Hence, the solar tracking system using such image methods are not suited to practical application. This study proposes an innovative image method to identify and evaluate the sun’s location. Eventually, we compare our method with that of using the Hough transform to find the sun.

## 2. Methods

The processes of our image method can be divided into the following three steps:

### 2.1. Transforming RGB to Grayscale Image

**Figure 1:**
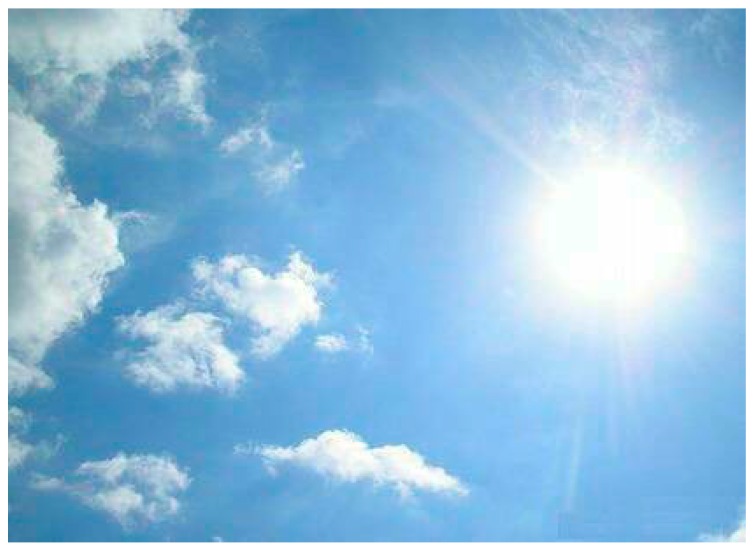
The color image of a sunny sky image.

**Figure 2:**
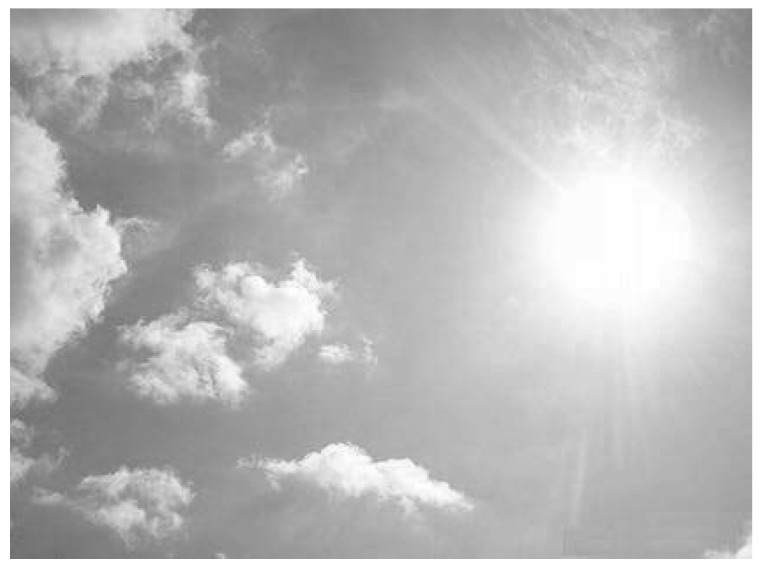
The grayscale image transformed from the RGB image.

The grayscale denotes the image, which transforms the format from the RGB color space to the YCbCr color space, and represents the brightness in the Y plane. Therefore, each pixel in the grayscale image indicates the luminance value. Its brightness range lies between 0~255. The conversion relationship between the luminance value and RGB value is listed in Equation (1) [18]. Y is the luma component and Cb and Cr are the blue-difference and red-difference chroma components. YCbCr color spaces are derived by a mathematical coordinate transformation, which is nonlinearly encoded based on the gamma-corrected RGB color space [19]. The nonlinear encoding can decrease the bandwidth or resolution required for transmission and storage. The form of YCbCr was defined for standard-definition television use in the ITU-R BT.601 (formerly CCIR 601) standard for use with digital component video [19]. Nowadays, Y can also be used as the grayscale image applied in digital image processing. Figure 1 shows the color sky image, and Figure 2 shows the grayscale image transformed from the RGB image.

(1) Y=0.299×R+0.587×G+0.114×B

### 2.2. Gaussian Smoothing Filter

**Figure 3:**
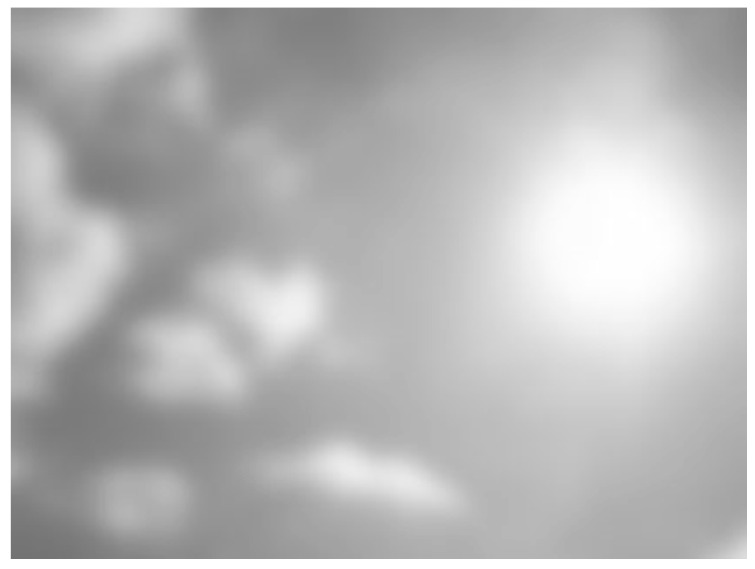
The grayscale image after the Gauss smoothing filter.

For some sudden high-frequency noises, a low-pass filter is necessary to remove them. In this paper, we used the Gaussian smoothing filter as the low-pass filter which uses a Gaussian function for calculating the transformation to apply to each pixel in the image. The Gaussian filtering G(*x*, *y*) in the following is used to blur the images and remove noise and extraneous details. The equation of the two-dimensional Gaussian function is expressed as follows:

(2) $$G(x,y)=\frac{1}{2\pi \sigma^{2}}e^{-\frac{x^{2}+y^{2}}{2\sigma^{2}}}$$

where *σ* is the standard deviation controlling the width of the cure shape, and *x* and *y* are the horizontal and vertical distances from the origin, respectively [18]. The distribution is assumed to have a mean of zero. A Gaussian blur effect is typically generated by convolving an image with a kernel of Gaussian values. Figure 3 shows the grayscale image after the use of the Gaussian smoothing filter.

### 2.3. Searching for the Brightest Region

At the key point of this research, we searched for the points with the maximum gray level value within this sky image. The arithmetic averages of the locations of such points were assumed to signify the sun centered areas. Therefore, we avoid the difficulty of finding the thresholding value while generating the binary image.

**Figure 4:**
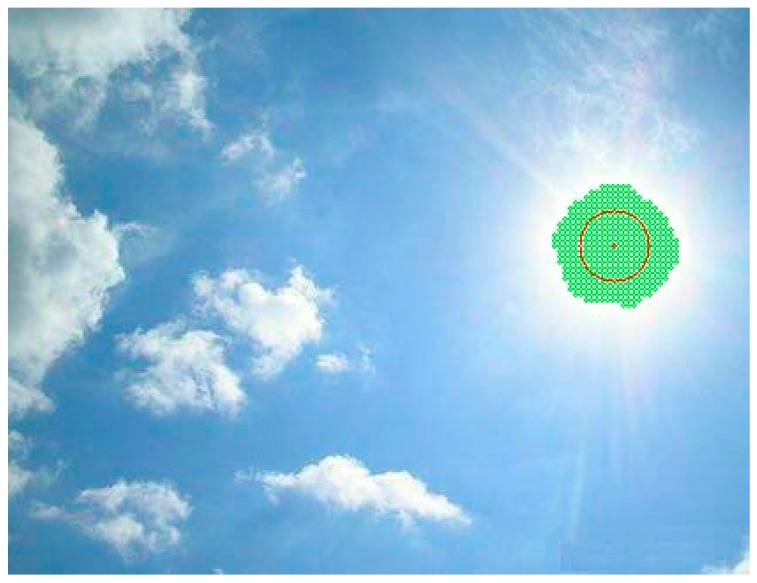
The green points have the maximum gray level value of 255. The red point is found by the arithmetic average of the x and y coordinates of the green points, respectively.

First, the sky image of a sunny day was captured and processed. Then, the points of the brightest region were found and marked as green points in Figure 4, where the maximum gray level value is 255. The red point in Figure 4 was found by the arithmetic average of the *x* and *y* coordinates of green points, respectively. It is assumed to be the sun’s center in our method. There is almost no deviation from the real sun’s center in the picture.

**Figure 5:**
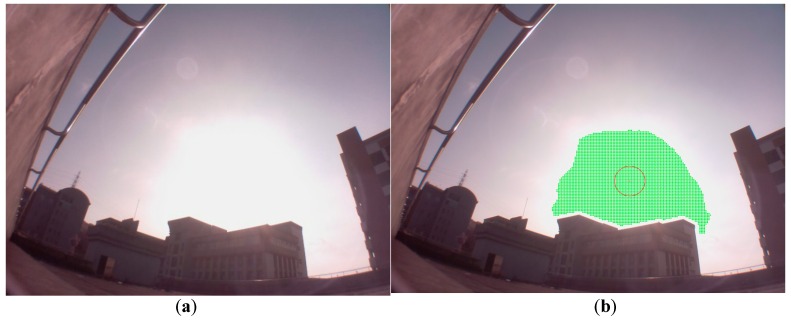
(**a**) The sun is sheltered by the building; (**b**) The evaluated center of the sun by the brightest region method is illustrated as a red point.

**Figure 6:**
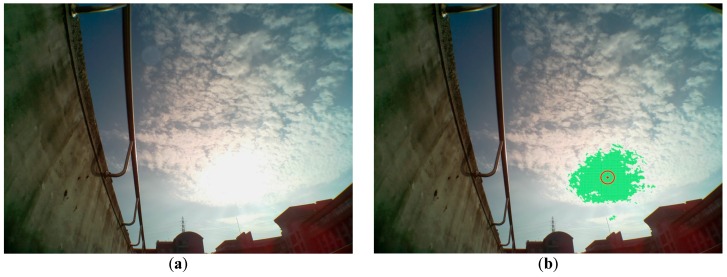
(**a**) The sun is shielded by the cloud; (**b**)The evaluated center of the sun by the brightest region method is illustrated as a red point.

Next, the image, where the sun is sheltered by the building, is demonstrated in Figure 5a. The evaluated center of the sun by the brightest region method is also illustrated as a red point in Figure 5b. The deviation ratio, which is defined as the distance from the real sun’s center divided by the diameter of the sun, is about 14.5% in Figure 5b. In Figure 6a, the sun is sheltered by the cloud, and the evaluated center of the sun by the brightest region method is illustrated as a red point in Figure 6b. The deviation ratio is about 6.7% in Figure 6b.

### 2.4. Comparison with the Image Method Using the Hough Transform

**Figure 7:**
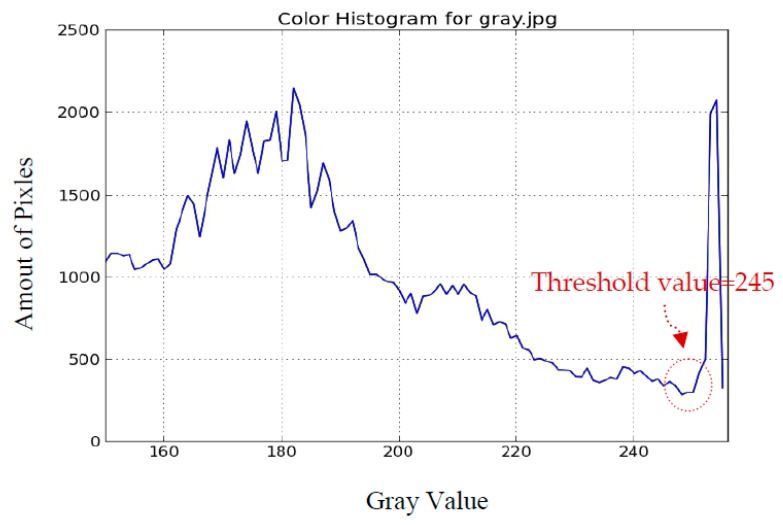
The histogram of Figure 1.

**Figure 8:**
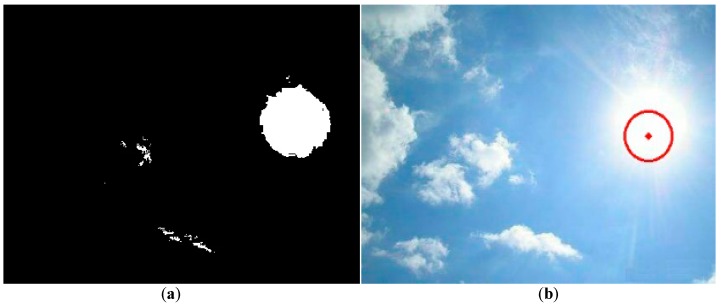
The binary image of Figure 1 is shown in (**a**); The evaluated center of the sun using the Hough transform method is shown as a diamond point in (**b**).

In this section, we compare our proposed method with another image method using the Hough transform to find the circular shape of sun and its position [16,20]. In image processing techniques, the Hough transform was applied to recognize the straight lines, circles, ellipses, and arbitrarily-shaped objects. In most image recognition techniques, including the Hough transform, the threshold value is one of the major concerns. An inappropriate threshold value may lead to the wrong recognition. We adopt a widely-applied method, histogram thresholding, to find the threshold value for generating the binary image [17]. The histogram of Figure 1 is shown in Figure 7. Basically, the pixel of the sun is brighter than its surroundings, and is attributed to the higher gray value. It is logical for us to choose the first value of the histogram as the threshold value to separate the sun from its surroundings. The threshold value of 245 is then manually chosen and applied to generate the binary image shown in Figure 8a. The white area in the right plane of Figure 8a appears like a circle, so the image separation using this threshold value is appropriate. The result using the Hough transform to recognize the circular sun and locate its position is demonstrated in Figure 8b as the diamond point. The deviation ratio is about 16.7%. Apparently, it is larger than the one using the brightest region method. 

**Figure 9:**
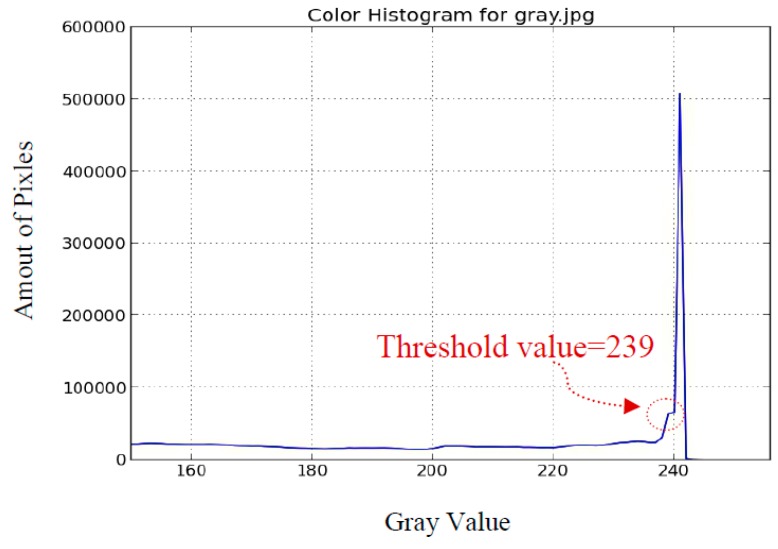
The histogram of the original image in Figure 5a.

**Figure 10:**
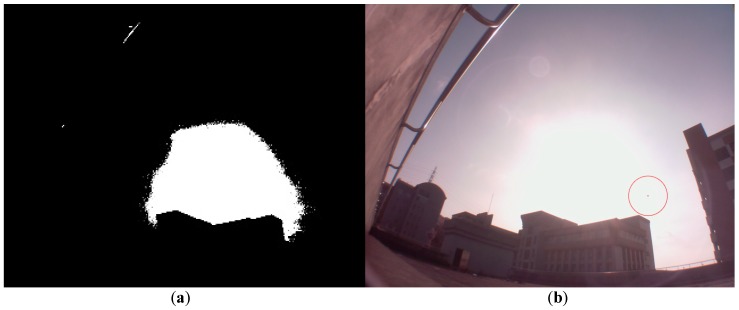
The related binary image is shown in (**a**); and the evaluated sun center by the Hough transform method is shown as a red point in (**b**).

We then use the Hough transform method to calculate the position of the sun center. The histogram of Figure 5a is shown in Figure 9; similarly, we manually choose 239 as the threshold value. The related binary image is shown in Figure 10a, and the white circle region is distorted because of the building. The evaluated sun center is shown as a red point in Figure 10b. The deviation ratio is about 33.6%, and is apparently higher than that by the brightest region method. The brightest region method reveals its superiority over the Hough transform method. Similar results are observed for Figure 6 because it is hard to obtain the circular-shaped object to recognize the sun.

## 3. Experiment and Results

**Figure 11:**
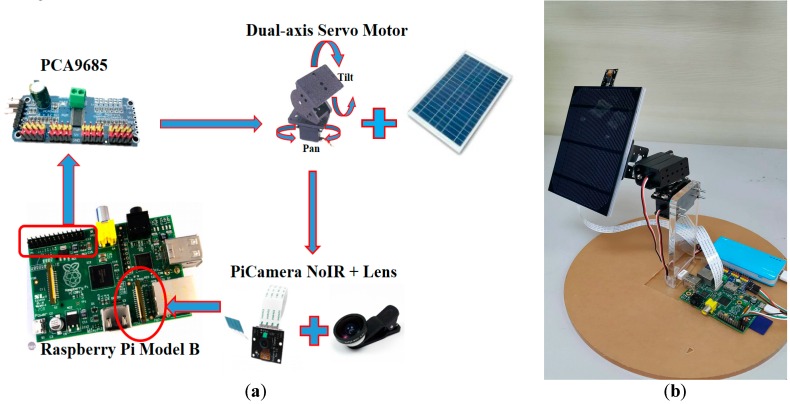
(**a**) The constituent parts of the solar tracking system are illustrated; (**b**) The completed system is shown.

The practical solar tracking system herein is composed of an embedded processor (Raspberry Pi) (RS Components, Corby, UK), a dual-axis servo motor, a pulse width modulation (PWM) servo driver (PCA 9685), a camera for the Pi ($20), and a small solar panel, as illustrated in Figure 11a. The camera board attaches to the Raspberry Pi via a 15-way ribbon cable. We need to get the cable the right way round, or the camera will not work. No optical filters, like ND (Neutral Density) filters, polarizing filters, or others were used on the camera of the Raspberry Pi. The major computer language in the system is Python, which is a widely used high-level, general-purpose, interpreted, dynamic programming language. Regarding the software of digital image processing, we used OpenCV (Open Source Computer Vision), which is a library of programming functions mainly aimed at real-time computer vision. The PWM signal used for controlling the motor can be generated by the GPIO (general-purpose input/output) pins of Raspberry Pi. The driver circuit (PCA 9685) can provide enough current for motors and acts as a bridge between the Raspberry Pi and the motors. Figure 11b shows the completed system, which is used to preliminarily verify our proposed method. The camera lens is vertically placed on the surface of the solar panel. In other words, if the evaluated sun center is located at the center of the picture, it means that the normal direction on the solar panel is aligned with that of the evaluated sun center. The original digital image of the RGB mode was captured by the camera of Raspberry Pi with a wide-angle lens. The camera size is around 25 × 24 × 9 mm^3^. Hence, it can be easily integrated into this solar power system. The still image resolution is 5 megapixels. Sensor resolution is 2592 × 1944 pixels. The image was captured on the top floor at Chaoyang University of Technology in Taiwan. 

**Figure 12:**
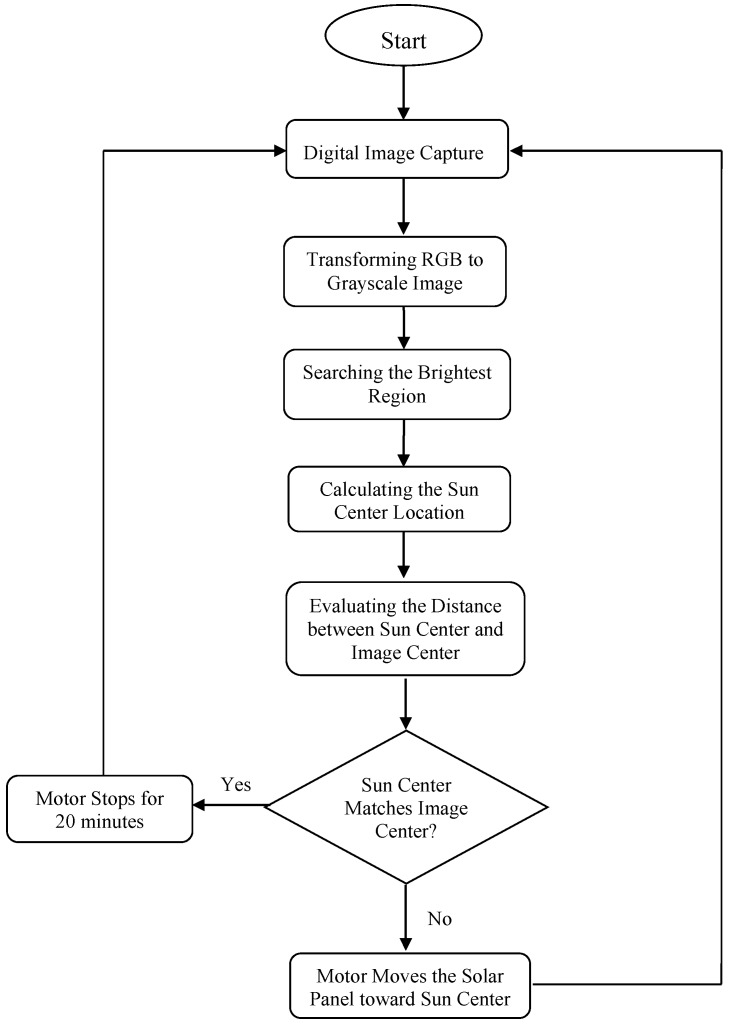
The operation flow of the solar tracking system.

The operation flow of the solar tracking system is demonstrated in Figure 12. If the evaluated sun center does not match the center of the picture, the tracking system will restart the image capture and identification procedure until the sun is located at the center of the picture. Hence, the tracking system possesses closed-loop feedback. If the evaluated sun center is located at the picture center, the motor will stop for twenty minutes before starting the next routine. 

**Figure 13:**
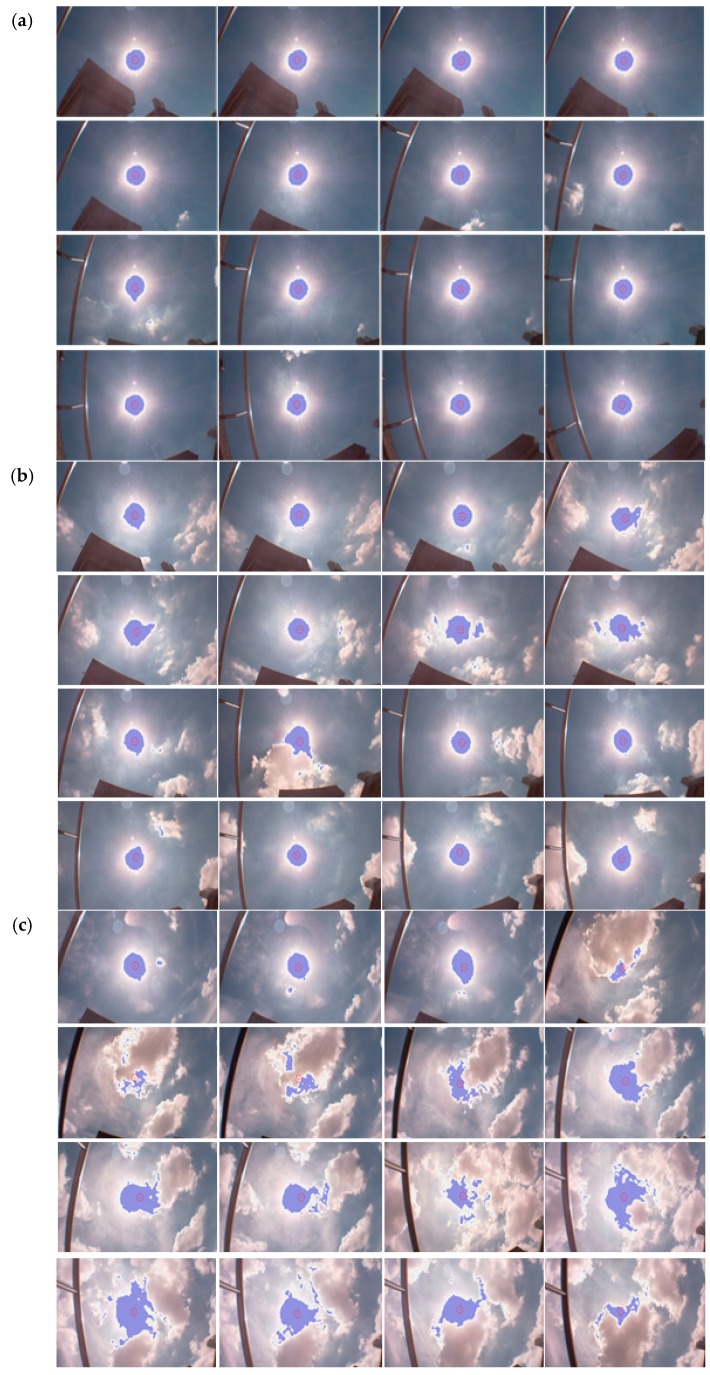
(**a**) The sky is clear, and the real sun center is marked with a red point. The brightest region is marked with blue points; (**b**) The sky is clear most of the time, but there are occasionally some clouds; (**c**) The sky is cloudy most of the time.

The captured images under various weather conditions, such as no clouds, few clouds, or some clouds, are recorded and discussed. In Figure 13a, the sky is clear, and the evaluated sun center approaches the real sun center well; the center is marked with a red point. The brightest region is marked with blue points. The handrail and surrounding buildings are also seen in the figure for reference position. Since the sun moves, the camera on the solar panel keeps tracking to move with the sun. Thus, the sun is always in the center of the picture; the handrail and surrounding buildings look like they are moving in the continuous pictures. In Figure 13b, the sky is clear most of the time, but sometimes there are clouds. We can find less deviation in the pictures with some clouds. In Figure 13c, the sky is cloudy most times. We can, thus, find some deviations from the real sun center in the pictures. Nevertheless, generally speaking, the system keeps good tracking of the sun.

## 4. Discussion

We have a single sun in the sky, and no other possible strong light source periodically appears in the sky. Therefore, the region where the sun is located signifies the brightest region in the sky and it is reasonable to find the sun’s position by identifying that region. Regarding the traditional image method to find the sun’s position, researchers focused on how to recognize the circular object in the sky, i.e., the sun. The key procedure in such a method is determining the optimum threshold value to generate the binary image. Sometimes it is difficult and complex to obtain the optimum threshold value by an automatic method, and should be done by a manual method; hence, it cannot be applied in a practical application. Even under some interference, there is no optimum value to generate a circular object in a binary image, and great deviations from the real sun center are observed. 

Regarding our brightest region method, it obviously precludes the threshold value problem. From the results we can know it is easy to obtain the accurate position of the sun in a clear sky. Under some slight interference resulting from less cloud, only a small deviation from the real sun center occurs. The evaluated sun center is always approximately within the sun region, and will not go out of control. However, when a large and thick cloud interferes, the bright side of the cloud will appear and the evaluated sun center is possible to be far from the real sun center. The problem under this situation still needs to be improved. The camera of the Raspberry Pi can not only be used in a solar tracking system, but can also be used in monitoring the weather conditions; it is important in managing and maintaining the solar energy system. In addition, the low cost and real-time operation are the advantages of our method. In summary, this novel image method provides a new way to overcome the disadvantages of the traditional image method for solar tracking, and makes the image method possible in real applications. However, we still face some challenges. For example, the clouds behave like space noises. Determining how to remove the noises by using a space filter and making the system more robust are worthy future endeavors.
